# LeishVet guidelines for the practical management of canine leishmaniosis

**DOI:** 10.1186/1756-3305-4-86

**Published:** 2011-05-20

**Authors:** Laia Solano-Gallego, Guadalupe Miró, Alek Koutinas, Luis Cardoso, Maria Grazia Pennisi, Luis Ferrer, Patrick Bourdeau, Gaetano Oliva, Gad Baneth

**Affiliations:** 1 Dep. Pathology and Infectious Diseases, Royal Veterinary College of London, UK; 2 Dpto. Sanidad Animal, Facultad de Veterinaria, Universidad Complutense de Madrid, Spain; 3 Companion Animal Clinic, Faculty of Veterinary Medicine, Aristotle University of Thessaloniki, Greece; 4 Dep. de Ciências Veterinárias, Universidade de Trás-os-Montes e Alto Douro, Portugal; 5 Dip.to Sanità Pubblica Veterinaria, Facoltà di Medicina Veterinaria, Polo Universitario Annunziata, Messina, Italy; 6 Dep. de Medicina i Cirurgia Animals, Universitat Autònoma de Barcelona, Spain; 7 Ecole Nationale Veterinaire Agroalimentaire et de l'alimentation Nantes-Atlantique (ONIRIS), France; 8 Dep. of Veterinary Clinical Sciences, Faculty of Veterinary Medicine, University of Naples Federico II, Italy; 9 School of Veterinary Medicine, Hebrew University, Israel

## Abstract

The LeishVet group has formed recommendations designed primarily to help the veterinary clinician in the management of canine leishmaniosis. The complexity of this zoonotic infection and the wide range of its clinical manifestations, from inapparent infection to severe disease, make the management of canine leishmaniosis challenging. The recommendations were constructed by combining a comprehensive review of evidence-based studies, extensive clinical experience and critical consensus opinion discussions. The guidelines presented here in a short version with graphical topic displays suggest standardized and rational approaches to the diagnosis, treatment, follow-up, control and prevention of canine leishmaniosis. A staging system that divides the disease into four stages is aimed at assisting the clinician in determining the appropriate therapy, forecasting prognosis, and implementing follow-up steps required for the management of the leishmaniosis patient.

## Background

Canine leishmaniosis (CanL) due to *Leishmania infantum *is a major global zoonosis potentially fatal to humans and dogs, which comprise the main reservoir of infection to humans [1]. CanL is endemic in more than 70 countries in the world. It is present in regions of southern Europe, Africa, Asia, South and Central America [2] and has been reported also in the United States of America (USA) [3]. It is also an important concern in non-endemic countries where imported sick or infected dogs constitute a veterinary and public health problem [4].

CanL is manifested by a broad spectrum of clinical signs and degrees of severity, and there is insufficient scientific agreement on the management of this disease [2]. LeishVet is a group of veterinary scientists from academic institutes in Europe and the Mediterranean basin with a main clinical and scientific interest in CanL. The main goal of LeishVet is to develop consensus recommendations that would represent the most current understanding of *L. infantum *infection in dogs based on recent evidence-based literature and clinical experience [2]. The objective of these guidelines is to help practitioners in the clinical management of CanL with emphasis on diagnosis, clinical staging, treatment, clinical monitoring, prognosis and prevention.

## Life cycle and transmission

**Figure 1:**
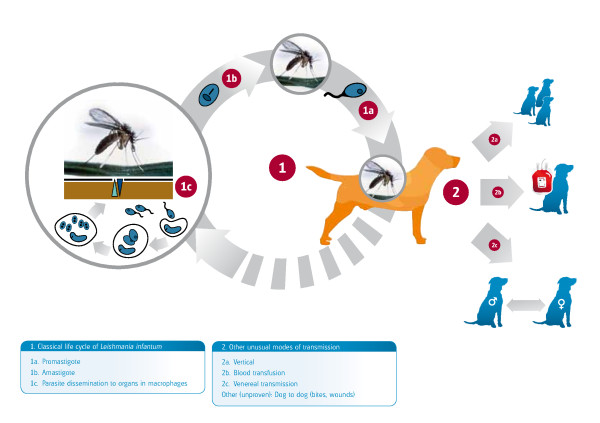
**The life cycle of *L. infantum *with indication of proven and unproven non-sandfly routes of transmission to dogs**.

**Figure 2:**
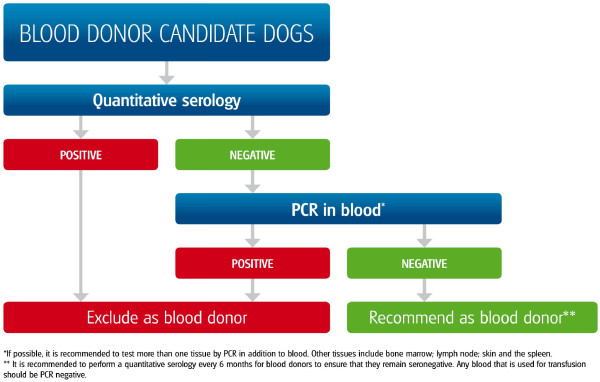
**Algorithm describing the selection of blood donors and exclusion of infected dogs**. Any dog infected will be excluded.

*Leishmania *completes its life cycle in two hosts, a phlebotomine sand fly vector, which transmits the flagellated infective promastigote form, and a mammal, where the intracellular amastigote form develops and replicates (Figure 1). Sand flies are the only arthropods that are adapted for biological transmission of *Leishmania*. The relatively low proportion of sand flies harbouring *L. infantum *(0.5 - 3%) is sufficient for maintaining the infection in endemic areas. Non-sand fly modes of transmission have also been described but their role in the natural history and epidemiology of leishmaniosis remains unclear (Figure 1). Proven modes of non-sand fly transmission include infection through transfused blood products [5] from blood donors which are carriers of infection [6,7], vertical [8-10] and venereal transmission [11]. The adequate selection of canine blood donors is of great importance for the prevention of *L. infantum *infection and recommendations on donor selection are graphically summarized in Figure 2. Suspected yet unproven modes of transmission include: 1) direct dog-to-dog transmission through bites or wounds, which could explain the presence of autochthonous CanL clinical cases [12] in non-endemic areas in the absence of apparent vectors, as described in foxhounds in the USA [13] or in breeding kennels in Europe [14], and 2) transmission by other hematophagous arthropods such as ticks and fleas [15-21] (Figure 1).

## Distribution and epidemiology

**Figure 3:**
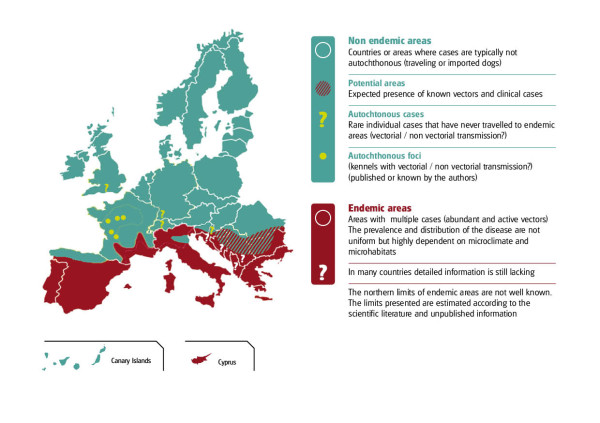
**The distribution of canine *L. infantum *infection in Europe**.

Socioeconomic and possible climate factors have led to changes in the distribution of CanL in Europe (Figure 3). *Leishmania infantum *infection has spread northward reaching the foothills of the Alps in northern Italy [22] and of the Pyrenees in France [14] and northern Spain [23]. The large numbers of dogs travelling to southern Europe or imported as companion animals from areas where CanL is endemic have increased the number of clinical cases reported in non endemic countries such as the United Kingdom [12] and Germany [24].

*Leishmania infantum *frequently follows an insidious and chronic pattern of infection [25]. Therefore, CanL is a disease in which infection does not equal clinical illness resulting in a high prevalence of subclinical infection [2,26].

**Figure 4:**
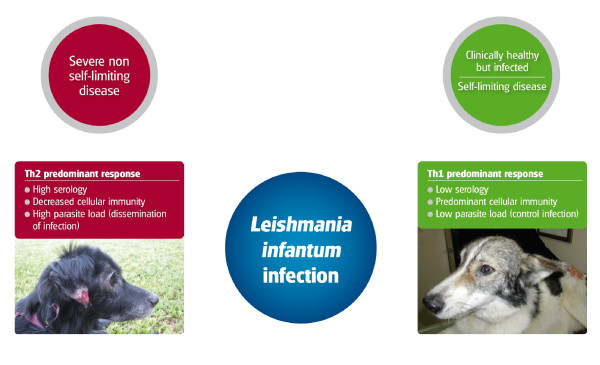
**Clinical manifestations and immunological characteristics of *L. infantum *infection in dogs**.

A broad range of immune responses and clinical manifestations have been described in CanL (Figure 4). Infection in dogs may be subclinical or manifested as a self-limiting disease, or a severe, and sometimes, fatal illness [27]. Subclinical infection is not necessarily permanent and factors such as immunosuppression or concomitant diseases could break the equilibrium and lead to the progression of clinical disease in dogs [2,27] as observed in humans coinfected with human immunodeficiency virus and *Leishmania *[28].

Several predisposing factors for the development of disease have been described including breed, age and genetic background. Some dog breeds such as the Boxer, Cocker Spaniel, Rottweiler and German Shepherd seem to be more susceptible to the development of disease [29,30], while others such as the Ibizian Hound rarely develop clinical signs of CanL [31]. The Slc11c1 (Solute carrier family 11 member a1) gene, formerly named N-RAMPI, and certain alleles of the MHC II genes have been associated with susceptibility to CanL [32,33]. Age seems to be an important factor. The distribution of the disease is bimodal, with the highest prevalence reported in dogs younger than 3 years and older than 8 years [34,35].

## Clinical manifestations and laboratory abnormalities

**Figure 5:**
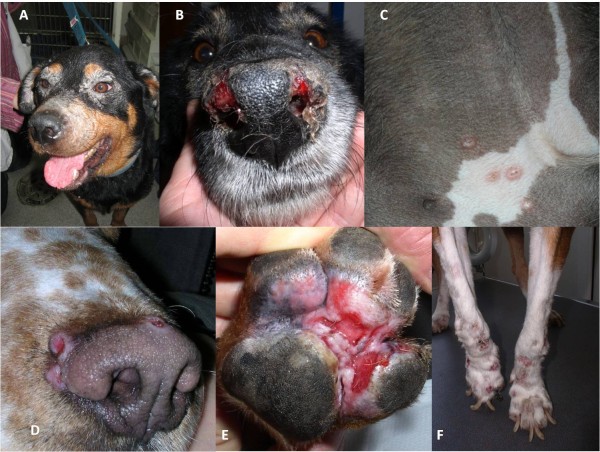
**Different patterns of cutaneous lesions in CanL**: A) Exfoliative periocular alopecia and blepharitis; B) Ulcerative nasal mucocutaneous lesions; C) Papular dermatitis in the inguinal region; D) Nodular crateriform lesions bordering the muzzle; E) Ulcerative erythematous lesions on the plantar surface of the paw and between pads; F) Onychogryphosis.

**Figure 6:**
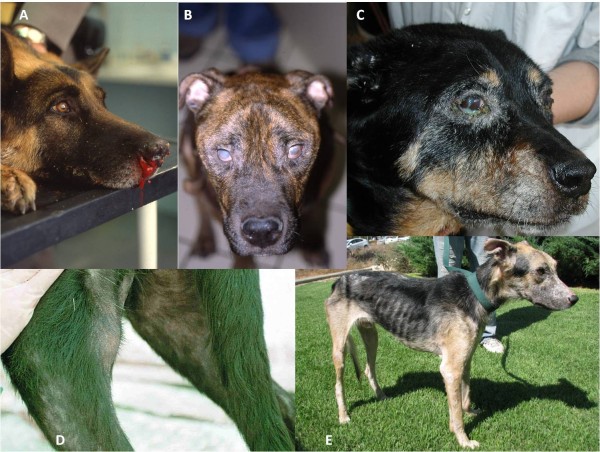
**Some clinical signs found in CanL**: A) Epistaxis; B) Bilateral uveitis and corneal opacity; C) Purulent conjunctivitis and blepharitis; D) Exfoliative alopecia in the rear leg and popliteal lymphadenomegaly; E) Marked cachexia and generalized exfoliative alopecia.

**Figure 7:**
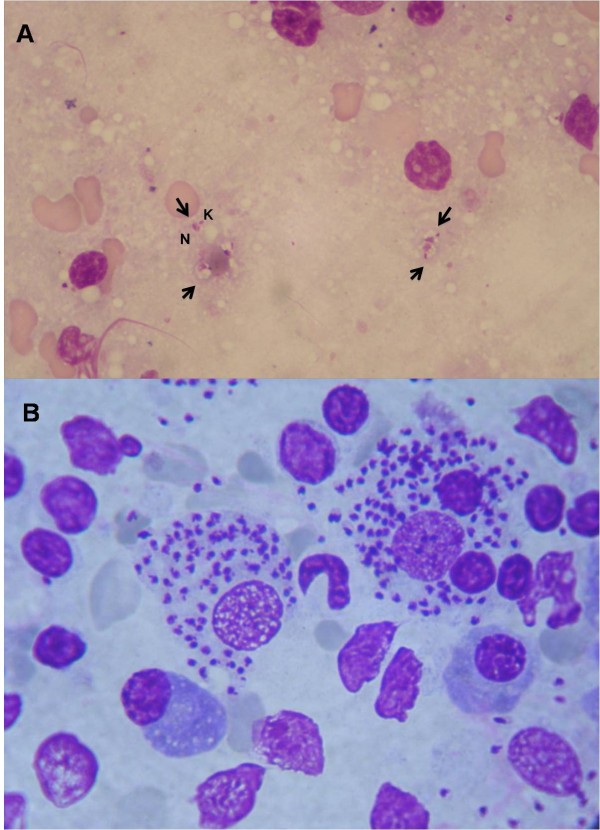
**Interpretation of cytology** A) Interpretation of cytology requires time and expertise for the detection of *Leishmania *amastigotes when parasites are in low numbers and freed from the cells. Note the nucleus (N) and the kinetoplast (K) of extracellular amastigotes (arrows) in a fine needle aspirate of a reactive lymph node from a dog with clinical leishmaniosis (x100, Diff-quick stain); B) High numbers of intracellular and extracellular *Leishmania *amastigotes in a fine needle aspirate of a reactive lymph node from a dog with clinical leishmaniosis (x100, modified Giemsa stain).

CanL is a systemic disease that may potentially involve any organ, tissue or body fluid and is manifested by nonspecific clinical signs. The most common clinical manifestations and clinicopathological abnormalities found in CanL are listed in Table 1[2,36,37]. Skin lesions are the most frequent manifestation among them (Figure 5) and may be seen along with other clinical signs or clinicopathological abnormalities. However, dogs can be presented with other clinical signs unrelated to cutaneous lesions as the main presenting complaint [36,37] (Figure 6). Renal disease may be the sole clinical manifestation of CanL and it can progress from mild proteinuria to the nephrotic syndrome or to an end stage renal disease. Chronic renal failure is a severe result of disease progression and the main cause of mortality due to CanL. Despite the high prevalence of renal pathology in infected dogs [38,39], renal azotemia is relatively an uncommon laboratory finding. The common pathological findings detected by cytology (Figure 7) or histology in CanL [40-43] are listed in Table 2. However, the variable and nonspecific clinical signs make the list of differential diagnoses wide and extensive.

**Table 1:** Clinical manifestations and laboratory abnormalities found in canine leishmaniosis due to *L. infantum*

Clinical manifestations	Laboratory abnormalities
**General** ○ Generalized lymphadenomegaly ○ Loss of body weight ○ Decreased or increased appetite ○ Lethargy ○ Mucous membranes pallor ○ Splenomegaly ○ Polyuria and polydypsia ○ Fever ○ Vomiting ○ Diarrhea (including chronic colitis)	**Serum proteins and electrophoretogram** ○ Hyperglobulinemia Polyclonal beta and/or gammaglobulinemia ○ Hypoalbuminemia ○ Decreased albumin/globulin ratio

**Cutaneous** ○ Non-pruritic exfoliative dermatitis with or without alopecia ○ Erosive-ulcerative dermatitis ○ Nodular dermatitis ○ Papular dermatitis ○ Pustular dermatitis ○ Onychogryphosis	**CBC/Hemostasis** ○ Mild to moderate non-regenerative anemia ○ Leukocytosis or leukopenia ○ Thrombocytopathy ○ Thrombocytopenia ○ Impaired secondary hemostasis and fibrinolysis

**Ocular** ○ Blepharitis (exfoliative, ulcerative, or nodular) and conjunctivitis (nodular) ○ Keratoconjunctivitis, either common or sicca ○ Anterior uveitis/Endophtalmitis	**Biochemical profile/urinalysis** ○ Mild to severe proteinuria ○ Renal azotemia ○ Elevated liver enzyme activities

**Other** ○ Mucocutaneous and mucosal ulcerative or nodular lesions (oral, genital and nasal) ○ Epistaxis ○ Lameness (erosive or non-erosive polyarthritis, osteomyelitis, polymyositis) ○ Atrophic masticatory myositis ○ Vascular disorders (systemic vasculitis, arterial thromboembolism) ○ Neurological disorders	

**Table 2:** Cytological and histopathological patterns suggestive of canine *L. infantum *infection found in organs or body fluids.

**Pathological findings in organs or body fluids**

✔ Macrophagic inflammation (granulomatous)
✔ Neutrophilic-macrophagic inflammation (pyogranulomatous)
✔ Lymphoplasmacytic inflammation
✔ Reactive hyperplasia in lymphoid organs
✔ No evidence or variable numbers of intracellular or extracellular *Leishmania *amastigotes

## Diagnosis

**Figure 8:**
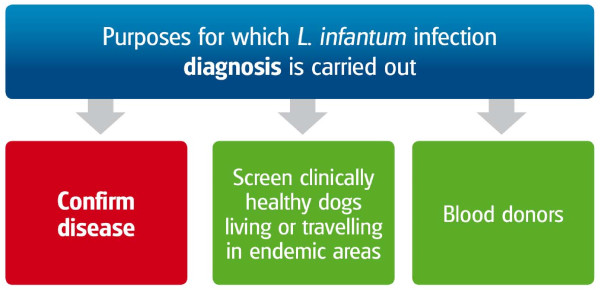
**The different purposes of CanL diagnosis**.

**Figure 9:**
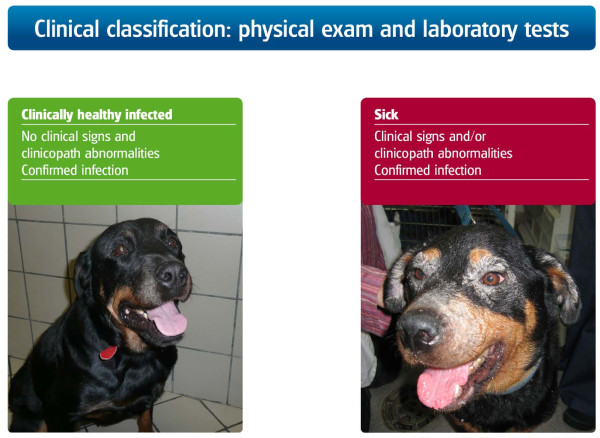
**Definition of *L. infantum*-infected but healthy *versus *sick dogs**. Dogs with clinical leishmaniosis are defined as those presenting clinical signs and/or clinicopathological abnormalities and having a confirmed *L. infantum *infection. Dogs with subclinical infection, or clinically healthy but infected dogs, are defined as those that present neither clinical signs on physical examination nor clinicopathological abnormalities by routine laboratory tests (CBC, biochemical profile and urinalysis) but have a confirmed *L. infantum *infection.

The purposes for which diagnosis of *L. infantum *infection is carried out are outlined in Figure 8. Due to these different diagnostic indications, it is important to separate *Leishmania *infection from disease and to apply different diagnostic techniques for each state. The definitions of sick *versus *clinically healthy infected dogs are shown in Figure 9 [27].

The diagnosis of CanL is complex as the clinical spectrum is broad and the range of clinicopathological abnormalities based on at least a complete blood count (CBC), biochemical profile and urinalysis can be both wide and non-specific. A thorough clinicopathological diagnostic approach needs to be adapted for each patient when assessing the suspicion of this disease. In addition, dogs with leishmaniosis might be co-infected with other vector borne diseases or suffering from other concomitant infectious or non-infectious diseases making the differential diagnoses more complicated and diverse. Therefore, based on the clinicopathological problem list, a differential diagnosis and specific diagnostic approach would be made for each patient.

**Figure 10:**
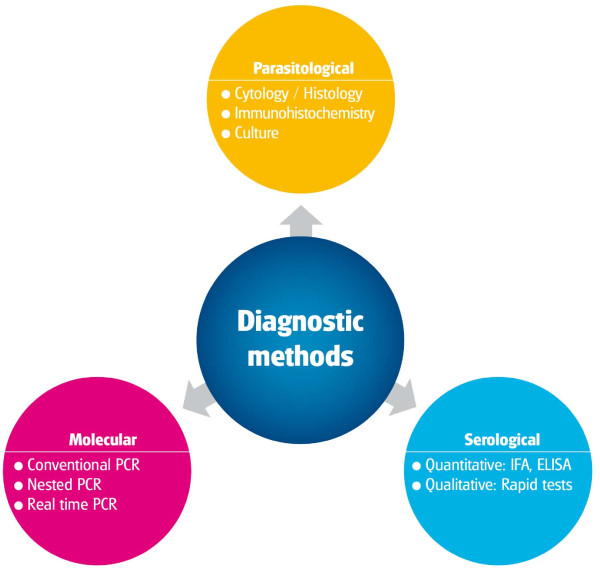
**The most common diagnostic methods for CanL**.

Different specific diagnostic methods have been described for the detection of *L. infantum *infection in dogs and these are shown in Figure 10. Valid diagnostic tests are essential for the detection of *Leishmania *infection in sick dogs although they lack 100% sensitivity and specificity [27]. The advantages and disadvantages of the different diagnostic methods are summarized in Table 3.

**Table 3:** Advantages and disadvantages of common diagnostic methods for the detection of *L. infantum *infection in dogs.

DIAGNOSTIC TECHNIQUES	ADVANTAGES	DISADVANTAGES
**SEROLOGY**	• Determination of antibody level which is essential for the diagnosis and establishing a prognosis	• Does not detect the actual presence of the *Leishmania *parasite • Serocrossreactions with trypanosomes

**QUALITATIVE**	• Rapid in-clinic test	• Provides only positive or negative result • Variable sensitivities and performance with risk of false negatives • A positive result needs to be further evaluated by a quantitative serology

**QUANTITATIVE** **(IFAT, ELISA)**	Determines the antibody level • High antibodies levels in the presence of compatible clinical signs and/or clinicopathological abnormalities are conclusive of clinical leishmaniosis	• Performance and accuracy of cut-off will depend on the laboratory • Differences between laboratories and poor standardization of techniques • Low antibody levels will require further work-up

**CYTOLOGY/HISTOPATHOLOGY**	Permits direct detection of the parasite itself and the type of pathological findings: - Pathological findings suspicious of infection - Allows exclusion of other differential diagnoses - Rapid and non invasive (cytology)	• Low sensitivity for the detection of *Leishmania *amastigotes in tissues or body fluids • Requires the performance of other diagnostic tests such as immunohistochemistry and/or PCR when parasites are not visualized • Does not reveal the immunological status of the dog • Needs expertise

**PCR**	• Allows the detection of leishmanial DNA • High sensitivity (kDNA) and specificity • Parasitic load quantification (if Real time-PCR)	• False positive results possible due to DNA contamination • Different standardization and techniques used by different diagnostic laboratories • Does not reveal immunological status • It cannot be performed as the sole diagnostic technique for the confirmation of the disease because a positive result confirms *Leishmania *infection but not disease

**PARASITE CULTURE**	• Permits the isolation of *Leishmania *parasites • Facilitates the isoenzymatic identification of the parasite	• Time-consuming and laborious diagnostic technique • It can require one month to provide a result • Performed only in research laboratories

**Figure 11:**
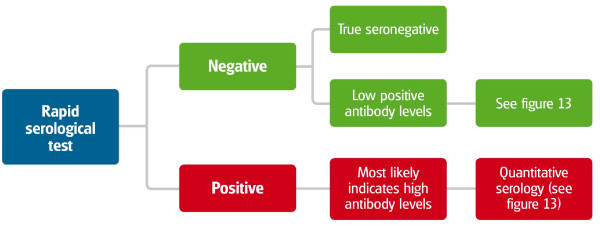
**Interpretation of serological qualitative rapid tests for CanL**.

The diagnosis of CanL can be made by the detection of specific serum antibodies (IgG) using preferably quantitative serological techniques, such as the immunofluorescence antibody test (IFAT) and enzyme-linked immunosorbent assay (ELISA). Immunochromatography-based assays are easy to use and provide rapid qualitative results on the spot, but their performance is still not optimal [44-46]. The interpretation of serological qualitative rapid tests is described in Figure 11. It is important to submit samples to a laboratory that runs quantitative serological assays and can provide an endpoint titer (IFAT) or an optical density reading (ELISA) and a classification of the level of antibodies [27].

**Figure 12:**
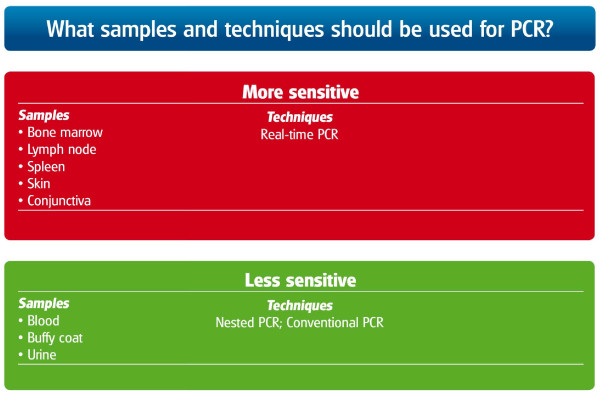
**Selection of tissues to be used for PCR and types of PCR techniques when suspecting CanL**.

Detection of *Leishmania *DNA in tissues by PCR allows sensitive and specific diagnosis of infection. PCR can be performed on DNA extracted from tissues, blood, body fluids or even from histopathologic specimens. The different sensitivities of tissues for the detection of *L. infantum *by PCR [27,47,48] and variable sensitivities of PCR techniques are listed in Figure 12. Assays based on the detection of kinetoplast DNA (kDNA) appear to be the most sensitive for direct detection in infected tissues [49,50]. Real-time PCR allows quantification of the *Leishmania *parasite load in the tissues of infected dogs, which is useful for the diagnosis and the follow-up during treatment [51,52]. It is important to highlight that information provided by PCR should not be separated from the data obtained from clinicopathological and serological evaluations.

**Figure 13:**
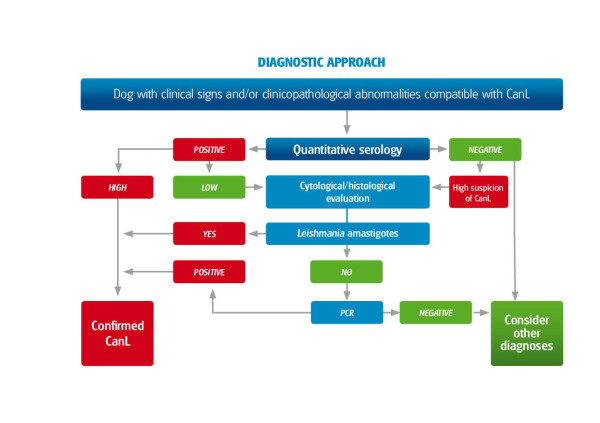
**Flow chart for the diagnostic approach to dogs with suspected clinical signs and/or clinicopathological abnormalities consistent with CanL**.

A high level of antibodies confirms the diagnosis of CanL in a dog with clinical signs and/or clinicopathological abnormalities compatible with leishmaniosis [53]. However, the presence of a low antibody level is not necessarily indicative of the disease and further work-up is necessary to confirm or exclude clinical leishmaniosis [27]. The diagnostic approach for sick dogs living in an endemic area is shown in Figure 13. The diagnostic approach for sick or healthy dogs living in a non-endemic area that have travelled to an endemic area, should include quantitative serology three months after the beginning of exposure in the endemic area.

## Clinical staging, treatment and prognosis

A system of four clinical stages based on clinical signs, clinicopathological abnormalities and serological status was proposed by the LeishVet group in an effort to cover the wide spectrum of clinical manifestations and degrees of severity found in CanL [27]. Different treatment protocols and prognoses are suggested for each clinical stage as described in Table 4.

**Table 4:** Clinical staging of canine leishmaniosis based on serological status, clinical signs, laboratory findings, and type of therapy and prognosis for each stage [27]

Clinical stages	Serology *	Clinical signs	Laboratory findings	Therapy	Prognosis
**Stage I Mild disease**	Negative to low positive antibody levels	Dogs with mild clinical signs such as peripheral lymphadenomegaly, or papular dermatitis	Usually no clinicopathological abnormalities observed Normal renal profile: creatinine < 1.4 mg/dl; non-proteinuric: UPC < 0.5	Scientific neglect/allopurinol or meglumine antimoniate or miltefosine/allopurinol + meglumine antimoniate or allopurinol + miltefosine**	Good

**Stage II Moderate disease**	Low to high positive antibody levels	Dogs, which apart from the signs listed in stage I, may present: diffuse or symmetrical cutaneous lesions such as exfoliative dermatitis/onychogryphosis, ulcerations (planum nasale, footpads, bony prominences, mucocutaneous junctions), anorexia, weight loss, fever, and epistaxis	Clinicopathological abnormalities such as mild non-regenerative anemia, hyperglobulinemia, hypoalbuminemia, serum hyperviscosity syndrome **Substages** a) Normal renal profile: creatinine < 1.4 mg/dl; non-proteinuric: UPC < 0.5 b) Creatinine <1.4 mg/dl; UPC = 0.5-1	Allopurinol + meglumine antimoniate or allopurinol+ miltefosine	Good to guarded

**Stage III Severe disease**	Medium to high positive antibody levels	Dogs, which apart from the signs listed in stages I and II, may present signs originating from immune-complex lesions: vasculitis, arthritis, uveitis and glomerulonephritis.	Clinicopathological abnormalities listed in stage II Chronic kidney disease (CKD) IRIS stage I with UPC > 1 or stage II (creatinine 1.4-2 mg/dl) [79]	Allopurinol + meglumine antimoniate or allopurinol + miltefosine Follow IRIS guidelines for CKD [80]	Guarded to poor

**Stage IV Very severe disease**	Medium to high positive antibody levels	Dogs with clinical signs listed in stage III. Pulmonary thromboembolism, or nephrotic syndrome and end stage renal disease	Clinicopathological abnormalities listed in stage II CKD IRIS stage III (creatinine 2-5 mg/dl) and stage IV (creatinine > 5 mg/dl) [79] Nephrotic syndrome: marked proteinuria UPC > 5	Allopurinol (alone) Follow IRIS guidelines for CKD [80]	Poor

*Dogs with negative to medium positive antibody levels should be confirmed as infected by other diagnostic techniques such as cytology, histology, immunohistochemistry or PCR. High levels of antibodies, defined as a 3-4 fold elevation above the cut off level of a well established reference laboratory, are conclusive of a diagnosis of CanL. **Dogs in stage I (mild disease) are likely to require less prolonged treatment with one or two combined drugs or alternatively monitoring with no treatment. However, there is limited information on dogs in this stage and, therefore, treatment options remain to be defined.

The most common drugs used for treatment of CanL, including dosage, combinations and side effects, are listed in Table 5. Several other candidate medications against CanL have been studied *in vitro *or in laboratory animals but rarely in controlled clinical trials and they are, therefore, currently not recommended for the routine treatment of CanL [27].

**Table 5:** Current treatment protocols for canine leishmaniosis [27]

Drugs	Dosages	Main side effects	References
Meglumine antimoniate*	75-100 mg/kg once a day or 40-75 mg/kg twice a day for 4 weeks, S.C.**	Potential nephrotoxicity Cutaneous abscesses/cellulitis	[52,55,57,81-83]

Miltefosine*	2 mg/kg/once a day for 28 days P.O.	Vomiting Diarrhea	[83-85]

Allopurinol	10 mg/kg twice a day for at least 6-12 months P.O.	Xanthine urolithiasis	[51,59,86-89]

*Registered for veterinary use in most European countries; both drugs are commonly recommended in combination with allopurinol.

P.O.: per os; S.C.: subcutaneous

**Treatment prolongation by 2-3 weeks may be considered if patient improvement is insufficient.

The clinical response to treatment of sick dogs can vary from poor to good depending on their overall initial clinicopathological status and their specific response to therapy. Dogs with renal insufficiency are expected to have a lower recovery rate in comparison to those without kidney compromise or only mild proteinuria. Therapy with antileishmanial drugs often leads to clinical cure [54] although treated dogs may continue to harbour the parasite and be infectious to sand flies, but to a lesser extent than pre-treatment [52,55-57].

The vast majority of dogs experience clinical improvement within the first month of therapy [51,52,58]; however, a longer period of therapy may be required for others before improvement is apparent. The frequencies of monitoring and clinicopathological parameters, including serology, to be followed up during treatment of CanL are summarized in Table 6.

**Table 6:** Treatment of canine leishmaniosis - recommended monitoring of clinicopathological parameters and serology including frequency of follow up [27].

Parameters	Frequency
Clinical history and complete physical examination Routine laboratory tests: Complete CBC, biochemical profile, serum electrophoresis (optional) and complete urinalysis including UPC in proteinuric dogs.	After the first month of treatment and then every 3-4 months during the first year. Later on, if the dog is fully recovered clinically with treatment, a recheck would be recommended every 6 months or once a year.

Serology*	Not before 6 months after initial treatment and every 6 months or once a year thereafter.

Real time PCR	Can optionally be carried out at the same time as serology. The full usefulness of this assay for follow up during treatment is currently undetermined.

*Some dogs present a significant decrease in antibody levels (more than a two-fold dilutions difference between the first and the following samples) associated with clinical improvement within 6 months to 1 year of treatment. Other dogs might not have a decrease in antibody levels despite clinical improvement. In contrast, a marked increase of antibody levels (more than two-fold elevation between monitoring samples) should be interpreted as a marker of relapse, especially in dogs following the discontinuation of treatment [27].

The length of allopurinol treatment depends on the severity of the disease, the clinical and parasitological response to treatment and the individual tolerance to this drug. Some extremely susceptible dogs never reach a point that would allow the discontinuation of allopurinol, while others are capable of controlling infection without the need for extremely lengthy treatment [27]. Allopurinol can be discontinued when the combination of the following criteria is made:

(1) The presence of complete physical and clinicopathological recovery evaluated by a thorough physical examination, CBC, full biochemistry panel and urinalysis.

(2) A marked decrease of antibody levels (to negative or borderline by a quantitative serological assay).

In addition, allopurinol might be discontinued if it is not possible to control or decrease the xanthinuria with low purine diets or by reducing the drug's dosage, to avoid the risk of urolithiasis, if massive xanthine crystalluria is present [59].

## Management of clinically healthy infected dogs in endemic areas

The management of clinically healthy infected dogs in areas where CanL is endemic is of great importance for practitioners.

The presence of *Leishmania *DNA in the blood or other tissues of clinically healthy dogs living in endemic areas indicates that these dogs harbour infection [26], but they may never develop clinical disease [60]. In contrast, a high positive antibody titer may indicate that an infected dog is heading towards the development of a widespread infection and future development of clinical disease [53]. Therefore, we recommend using serology alone or the combination of serology with PCR for screening healthy dogs. It is recommended to avoid screening clinically healthy dogs only by PCR.

**Figure 14:**
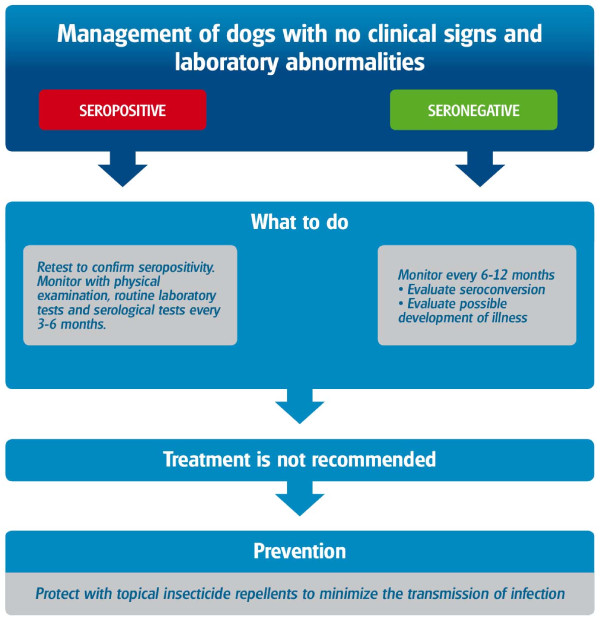
**Management of *Leishmania-*seropositive but clinically healthy dogs and PCR-positive but seronegative dogs**. Clinically healthy but seropositive dogs would normally present with low antibody titers and should be confirmed by retesting. Confirmed seropositive dogs should be monitored with physical examinations, routine laboratory tests and serological tests on a regular basis every 3-6 months to assess the progression of infection towards disease.

Healthy dogs should be screened for *Leishmania *antibodies as an initial indication for the presence of infection if [27]:

1. They are scheduled to travel or be exported to non-endemic areas (Figure 14)

2. They serve as blood donors (Figure 2)

3. Their owners wish to have them monitored at least every 12 months for early detection of infection and the potential to develop disease.

PCR should be used for the second above indication and as an ancillary test for the first and third above indications.

The management of a clinically healthy seropositive dog and a clinically healthy seronegative and PCR-positive dog is summarized in Figure 14.

## Prevention

Current preventative measures are mainly based on the use of veterinary registered products containing synthetic pyrethroids, permethrin, or deltamethrin with a repellent effect against sand flies whose efficacy has been demonstrated both experimentally [61-63] and in field studies [64-68]. These products are available in spot on formulation or in a collar form and they reduce the risk of new infections and the biting of sand flies on already infected dogs [64-68]. Other measures useful in the prevention of sand fly bites include [69,70]: 1) keeping the dog indoors during the sand fly season from dusk to dawn; 2) reducing microhabitats favourable to sand flies such as piles of wood and stones in the vicinity of the house and in other locations where dogs spend time; 3) usage of indoor insecticide treatment [27].

Long-acting topical insecticides evaluated in several published field studies [64-68] should be applied to dogs living or travelling to endemic areas as follows:

- Permethrin/Imidacloprid spot on formulation: Treatment provides repellent (anti feeding) activity against sand flies (*P. perniciosus*) for three weeks [61]. Repeat administration every 3 weeks. It should be applied at least 2 days before travelling

- Deltamethrin collars: Control of feeding by phlebotomine sand flies (*P. perniciosus*) for a period of 5-6 months [63]. Replace collar every 5-6 months. It should be applied at least 1-2 weeks before travelling.

Veterinarians and dog owners are advised to carefully check the product's label recommendations and follow the manufacturer's instructions for the correct application and frequency of reapplication. Client education on the maintenance of an appropriate insecticide [27] throughout the period of sand fly activity in the Mediterranean basin (April-November) is also crucial for the protection of dogs [71].

Purified *Leishmania *fraction vaccines appear currently to be the most effective and promising vaccines for dogs. These include the ''fucose mannose ligand'' (FML)-based vaccine [72,73] and an excreted/secreted antigen purified from specific-medium culture supernatant of *L. infantum *based vaccine [74]. The FML-based vaccine is currently available commercially in Brazil. The same vaccine has also been proposed as a transmission-blocking vaccine [75]. Recently, another vaccine which contains the recombinant A2 protein and saponin as adjuvant has also been approved in Brazil [76]. In Europe, a different vaccine based on cultured *L. infantum *purified excreted/secreted antigens has been approved for vaccination of dogs [74].

The future for CanL control should be an integrated approach to prevention including vaccination against *L. infantum *with an effective canine vaccine and the use of long-acting topical insecticide applications. A vaccine would prevent the establishment of infection introduced by the bites of those sand flies that escape the insecticide effect [50].

## Public health considerations

In Southern Europe, human visceral leishmaniosis caused by *L. infantum *is a zoonotic disease that affects young children or adults suffering from the Acquired Immune Deficiency Syndrome (AIDS) or immunosuppressive conditions [77,78]. Dogs are considered the most important peridomestic reservoir of *L. infantum *infection for humans. However, the ownership of an infected dog does not appear to greatly increase the risk of disease in the family when transmission is already present in the region [50].

## Conclusions

The complexity of CanL and the wide range of its clinical manifestations, from inapparent infection to severe disease, make the management of CanL challenging. Diagnosis is performed based on clinicopathological manifestations and by confirmation of infection using mainly serological and molecular techniques. A staging system that divides the disease into four stages is aimed at assisting the clinician in determining the appropriate therapy, forecasting prognosis, and implementing follow-up steps required for the management of the leishmaniosis patient. Prevention should be an integrated approach including vaccination against *L. infantum *with an effective canine vaccine and the application of a topical insecticide.

## List of abbreviations

AIDS: Acquired Immune Deficiency Syndrome; CanL: Canine leishmaniosis; CBC: Complete blood count; CKD: Chronic kidney disease); DNA: Deoxyribonucleic acid; ELISA: Enzyme-linked immunosorbent assay; FML: Fucose mannose ligand; IFAT: Immunofluorescence antibody test; IgG: Immunoglobulin G; IRIS: International Renal Interest Society; kDNA: Kinetoplast DNA; MHC II: Major histocompatibility complex type II; N-RAMPI: Natural Resistance-associated Macrophage Protein one; PCR: Polymerase chain reaction; P.O.: per os; S.C.: subcutaneous; Slc11c1: Solute carrier family 11 member a1; UPC: Urinary protein creatinine ratio; USA: United States of America

## Competing interests

The authors declare that they have no competing interests.

## Authors' contributions

LSG, GM, AK, LC, MGP, LF, PB, GO and GB participated in the formation of the manuscript's content. LSG coordinated the preparation and writing of the manuscript. All authors contributed to helpful discussions, read and approved the final manuscript.
